# Infectious disease screening in asylum seekers: range, coverage and economic evaluation in Germany, 2015

**DOI:** 10.2807/1560-7917.ES.2017.22.40.16-00677

**Published:** 2017-10-05

**Authors:** Kayvan Bozorgmehr, Katharina Wahedi, Stefan Noest, Joachim Szecsenyi, Oliver Razum

**Affiliations:** 1 Department of General Practice and Health Services Research, University Hospital Heidelberg, Heidelberg, Germany; 2 Department of Epidemiology & International Public Health, School of Public Health, Bielefeld University, Bielefeld, Germany

**Keywords:** evidence-based medicine, infection control, policy, screening, migration, refugees, economic evaluation, asylum seekers

## Abstract

Screening asylum seekers for infectious diseases is widely performed, but economic evaluations of such are scarce. We performed a policy analysis and economic evaluation of such screening in Germany, and analysed the effect of screening policies on cost differences between federal states. Of the 16 states, screening was compulsory for tuberculosis (TB) in asylum seekers ≥ 16 years of age in all states as well as in children < 16 years of age and pregnant women in six states, hepatitis B and enteropathogens in three, syphilis in two and human immunodeficiency virus (HIV) in one state. Of 441,899 asylum seekers, 88.0% were screened for TB, 22.9% for enteropathogens, 16.9% for hepatitis B, 13.1% for syphilis and 11.3% for HIV. The total costs for compulsory screening in 2015 were 10.3 million euros (EUR). Costs per case were highest for infections with *Shigella* spp. (80,200 EUR), *Salmonella* spp. (8,000 EUR), TB in those ≥ 16 years of age (5,300 EUR) and syphilis (1,150 EUR). States with extended screening had per capita costs 2.84 times those of states that exclusively screened for TB in asylum seekers ≥ 16 years of age (p < 0.0001, 95% confidence interval (CI): 1.96–4.10). Screening practices in Germany entailed high costs; evidence-based approaches to infectious disease screening are needed.

## Introduction

Upon-entry medical screening of asylum seekers is a cornerstone of infectious disease control programmes in nearly all countries of the European Union (EU) [1,2]. The content of medical screening programmes, as well as their legal obligation for asylum seekers, varies considerably between countries [1,2]. Screening programmes may also differ in focus and aim, with some focusing on infectious disease control and the protection of public health [3,4] and others focusing on the prevention of disease spread in community housing [5] or the early identification of vulnerable groups’ needs [6].

Any screening programme, however, needs to follow Wilson and Jungner’s classic screening criteria, which consider the public health importance of the condition, the screened population’s access to an accepted treatment, the economic balance between the cost of case-finding and the expenditure on medical care as a whole, as well as case-finding as a continuing process [7]. A synthesis of screening criteria proposed over the past 40 years also demands that screening objectives be defined at the outset, that scientific evidence of screening effectiveness be integrated into clinical services, quality assurance, and programme management and evaluation [8]. Such criteria are especially important when screening is mandatory [7].

Many countries, including Germany, are currently implementing a pragmatic approach [5,9] by defining screening criteria through legislation, experience or expert recommendations. Some have also created evidence-based guidelines. One very comprehensive set of evidence-based guidelines was formulated by the Canadian Collaboration for Immigrant and Refugee Health; their guidelines pertain to four areas of migrant screening, infectious disease, mental health, chronic and non-communicable disease and women’s health, and take differences in disease prevalence and perceived needs between migrant populations into account [6]. A scientific panel facilitated by the European Centre for Disease Prevention and Control (ECDC) is also developing evidence-based guidelines [9].

Germany is one of the main countries receiving asylum seekers in the EU [10]. Even though national law defines a limited set of screening measures to be conducted, medical screening policies are predominantly governed by federal state law. The only screening measure governed by national law (§62 of the Asylum-Law in combination with §36 of the Infection Protection Act) is a compulsory chest X-ray examination in asylum seekers ≥ 16 years of age to identify active pulmonary tuberculosis (TB). All arriving asylum seekers are registered with an identification number and are quasi-randomly allocated to one of the 16 federal states by means of an administrative quota (Königsteiner Schlüssel) [11] that aims to achieve a weighted distribution of asylum seekers based on tax income and population size of respective federal states in order to achieve ‘fairness’ in the resulting economic burden [12]. Here they undergo a mandatory, upon-entry medical screening according to the state’s policies. Upon-entry screening for TB in children (< 16 years of age) and pregnant women is governed by differing policies in each of the 16 federal states [13]. Detailed information on the content of federal state-level medical screening policies is not always publicly accessible. Furthermore, yields of screening are not available in many federal states due to a lack of denominator data and limitations of the health information system [14]. Furthermore, screening programmes under national and state-level law lack systematic integration of quality assurance and evaluation measures, so that little is known about their effectiveness [13].

Decentralising the governance of screening programmes to the federal state level leads to considerable heterogeneity of screening measure content and voluntariness within Germany. Due to the absence of nation-wide binding standards, a substantial heterogeneity also exists within and between federal states with respect to contracting, purchasing and re-imbursement schemes for healthcare provision to asylum seekers, including medical screening. While some federal states delegate mandatory medical screening to public health services, others contract physicians of statutory sickness funds, physicians operating privately, hospitals, non-governmental organizations or commercial providers.

Due to this heterogeneity and the difficulties in obtaining detailed information, previous studies have failed to generate an overview of screening policies’ content in Germany [1] or provided an incomplete picture of performed measures [15]. Country-wide studies broken down by federal states have not yet been conducted on the coverage, yields and costs of screening programmes. An analysis of these aspects could provide valuable insights on screening strategies. The quasi-random distribution of asylum seekers to federal states with heterogeneous screening policies allows for an analysis of differences in costs between states that minimises influences attributable to individual-level differences. This information could provide important lessons for other regions or countries.

In this study, we estimated the coverage and costs relative to expected yields of medical screening programmes currently operating in the 16 German federal states and compared the effect of screening policies on differences in screening programme costs between states.

## Methods

### Analysis of the range of screening policies

In a previous study we performed a nation-wide assessment of healthcare provision to asylum seekers between June and October 2015 [13]. As part of the previous study, to determine the content of medical screening programmes, we requested written policy documents governing federal state level medical screening policies from health authorities. For this study, we used these policy documents and where these did not exist, we used publicly available information such as parliamentary enquiries and that obtained from semi-structured qualitative interviews with 36 representatives and heads of public health authorities from all 16 federal states. As part of this study, we performed a content analysis using MaxQDA (version 12), coding the content of screening policies (e.g. hepatitis B, syphilis, human immunodeficiency virus (HIV), TB, the eligibility criteria for each screening measure and information on whether measures were defined as compulsory or voluntary. We categorised the coded information into four broad themes, (i) type of disease, (ii) diagnostic tests used, (iii) eligible population groups and (iv) voluntariness, and extracted these in a code-matrix for quantitative analysis of coverage, costs and screening outcome.

### Estimating coverage

We created a binary variable (1/0) for each diagnostic measure (T) conducted by the federal states on a compulsory basis. To estimate the proportion of asylum seekers in a federal state affected by the diagnostic measures (i.e. the proportion of people subject to a measure among those eligible to undergo a measure), we weighted the binary variable by multiplying it with the administrative quota (w) for the year 2015 taken from the Joint Science Conference [11]. We used aggregated, representative socio-demographic data of the Federal Office for Migration and Refugees (BAMF) on 441,899 first-time applicants for asylum in the year 2015 [16] to estimate the total number of asylum seekers eligible to undergo the different diagnostic measures. According to BAMF, a first-time applicant for asylum is considered a registered person who has applied for asylum and whom the decision is pending. Age groupings for compulsory tests were as follows: 0–5 years of age, 6–15 years of age, and 16 years of age and above. Due to the lack of reliable national data, we assumed the prevalence of pregnant women among all newly arrived female asylum seekers to be 1% of female first applicants based on narrative experiences of care providers in reception centres.

We calculated the absolute number of asylum seekers covered by mandatory diagnostic tests at federal state-level as:

$$C_{j,Tx}=T_{x}\times w_{j}\times N_{eligibilitygroupTx}$$

where the coverage *C* is the number of individuals affected by a mandatory diagnostic test in state *j*. *T* is the binary variable with values for presence (1) or absence (0) of a mandatory test for disease *x* (e.g. hepatitis B, HIV or TB) and *w* is the weight according to administrative quota in state *j.* *N* is the total, country-level number of asylum seekers falling under the eligibility criteria of the test for disease *x*. We then calculated the total, country-level coverage of asylum seekers *C_total_* for each diagnostic test across the 16 federal states as:

$$\sum_{j=1}^{16}C_{j}=C_{total}$$

### Estimating costs

We estimated the monetary costs for each mandatory diagnostic measure using market values for each measure from a healthcare perspective. There are two main reimbursement schemes for health service providers in Germany: (i) statutory insurance fees (Einheitlicher Bewertungsmaßstab, EBM), which involves reimbursement according to fees negotiated by statutory sickness funds and the association of statutory sickness fund physicians [17]; (ii) private fees (Gebührenordnung der Ärzte, GOÄ), which involves reimbursement according to fees set by physicians for patients with private insurance or without any type of insurance [18]. The minimum price for a given service is usually higher than the statutory insurance fee by a non-constant factor.

Due to the lack of data on federal state expenditure for medical screening of asylum seekers, we used unit costs of statutory insurance fees for the different diagnostic measures in order to obtain a conservative (lower-bound) estimate of the medical screening programme costs in each federal state. The total cost *TC* for each diagnostic measure *T* of disease *x* in state *j* was thus calculated as:

$$TC_{j,Tx}=\text{unitcost }\times \text{​}T_{x}\times n_{eligibilitygroup,j}$$

where *unitcost* in EUR is determined by statutory insurance fees and *n* refers to the number of individuals screened in respective eligibility groups in state *j*. Where tests were not specified in screening policies, we used the most common diagnostic test to estimate costs for interferon gamma release assays (IGRAs).

We calculated the total costs of all medical screening measures in each state as the sum of costs for all diagnostic tests performed in that state. We then calculated, stratified by category of screening policy as defined below, the average total costs of medical screening in asylum seekers as the total costs divided by the number of federal states. Per capita costs of screening were calculated as the total costs divided by the total number of asylum seekers in each state or category of screening policy.

### Measures of screening outcome

We used the yield of screening programmes, i.e. the number of cases identified through screening for a specific disease divided by the total number of asylum seekers screened for that disease, as a measure of screening outcome. Given that yields of screening of asylum seekers under national law are not reported on a regular basis in Germany, we used publicly reported yields of the screening programme in Bavaria [19,20], the federal state with the second highest numbers of asylum seekers, to estimate the expected nation-wide number of identified cases of screening for hepatitis B, HIV and enteropathogens (*Shigella* spp., *Salmonella* spp. and intestinal parasites). Yields of the mandatory chest X-ray to rule out active TB were taken from a systematic review and meta-analysis of German studies [21] that found the yield of TB screening to be in line with a meta-analysis of international studies [22].

We used the yields to estimate the number of cases of respective diseases identified by screening programmes in a given state according to the following equation:

$$n_{cases,j,x}=Yield_{Tx}\times {\text{Τ}}_{x}\times n_{e}{}_{ligibilitygroup,j}$$

where *n_cases_* is the number of cases of disease *x* in state *j*, *Yield_Tx_* is the yield of the diagnostic measure obtained from the above sources and *n_eligibilitygroup_* is the total number of asylum seekers in state *j* fulfilling the eligibility criteria for the test.

### Economic evaluation of costs relative to screening outcomes

We evaluated the costs per identified case of each disease from a healthcare perspective. Costs per identified case of disease *x* were calculated as total costs for each diagnostic measure *TC_Tx_* divided by the estimated total, country-level number of cases *N_cases,x_* identified by each test:

$${\text{Cost per case}}_{x}=TC_{Tx}÷N_{cases,x}$$

### Comparing the effect of screening policies on differences in costs between states

To compare the effect of screening policies on differences in costs between states, we categorised the 16 federal states into three groups according to their screening policies:

- Category A: States limiting compulsory screening to the minimum required by national law, i.e. screening for TB in asylum seekers ≥ 16 years of age by means of a chest X-ray.
- Category B: States performing an extended mandatory screening for TB in children < 16 years and/or pregnant women by means of diagnostic tests such as tuberculin skin tests (TST) and/or IGRA in addition to the measures described in Category A.
- Category C: States performing an extended mandatory screening for infectious diseases in addition to the measures mentioned in Category A or B.

Differences in average total costs and per capita costs of asylum seeker medical screening programmes by group of screening policy (Category A, B, C) at federal state-level were assessed using scatter plots with fitted linear regression lines. The absolute effect of screening policies (Category A, B, C) on per capita costs was then quantified by means of linear regression analysis according to:

$$y=\beta_{0}+\beta_{1}\times SCREENPOLICY+\varepsilon$$

where *y* is per capita cost in EUR, ε is the error term with normal distribution and zero mean, *β_0_* is the constant and *β_1_* is the regression coefficient for states in respective categories (Category A, B, C) of the variable *SCREENPOLICY,* interpreted as the absolute average difference in per capita costs between federal states in Category B or C and the reference states in Category A.

The relative effect was quantified by calculating the exponential of the regression coefficient of screening policies (exp(*β_1_*)) obtained from linear regression models using the natural logarithm of per capita costs of medical screening programmes (log(*y*)) as outcome.

### Sensitivity analyses

We performed sensitivity analyses using private fees as unit costs as per the equation under ‘Estimating costs’ to assess the possible range of total costs, per capita costs of screening and costs per identified case of disease if full private arrangements were chosen by states. We also used private fees to calculate *y* as per the equation under ‘Comparing the effect of screening policies on differences in costs between states’ to assess the absolute and relative effects of policies on per capita costs in regression analyses.

### Statistical analysis

We used bootstrapping techniques with up to 1,000 replications to calculate standard errors and 95% confidence intervals (CI) for mean total costs. In regression models, we calculated robust standard errors to account for the clustering of per capita costs in federal states. Microsoft Excel was used for data management and all analyses were performed using Stata version 12.1.

## Results

### Screening tests and coverage

The range of screening tests conducted on a compulsory basis comprised screening for TB in asylum seekers ≥ 16 years of age, screening for TB in children < 16 years of age and/or pregnant women, and screening for hepatitis B, HIV, syphilis and enteropathogens in all age groups (Table 1).

**Table 1:** Mandatory screening measures by eligibility and affected asylum seekers, Germany, 2015 (n = 441,899 first-time applicant asylum seekers)

Infectious disease screened for	Number of federal states performing screening	Eligible population of first-time applicants	Number of asylum seekers eligible	%	Percentage of eligible asylum seekers^a^ affected by screening measure	Number of asylum seekers affected by screening measure	%
Hepatitis B	3	≥ 16 years of age	324,796	73.5	23.0	74,568	16.9
TB (all groups)	NA	NA	NA		NA	389,052	88.0
TB in non-pregnant adults	16	≥ 16 years of age	323,958	73.3	100	323,958	73.3
TB in children	6	< 16 years of age	117,103	26.5	55.1	64,551	14.6
TB in pregnant women	6	≥ 16 years of age and pregnant	838	0.19	64.7	542	0.1
Stool examination^b^	3	All ages	441,899	100	22.9	101,255	22.9
Syphilis	2	≥ 16 years of age	324,796	73.5	17.9	58,002	13.1
HIV	1	≥ 16 years of age	324,796	73.5	15.3	49,793	11.3

NA: not applicable; TB: tuberculosis.

^a^ Percentage of eligible asylum seekers was calculated as the sum of administrative quota of those states which perform respective screening measures.

**^b^** Stool examination for enteropathogens, i.e. *Salmonella* spp. (typhus, para-typhus), *Shigella* spp., and worms.

Based on our estimates, the screening measure that affected the highest proportion of asylum seekers among the total population (n = 441,899) was screening for TB (88.0%), followed by stool examinations for enteropathogens (22.9%) and serological screening for hepatitis B (16.9%).

**Figure 1:**
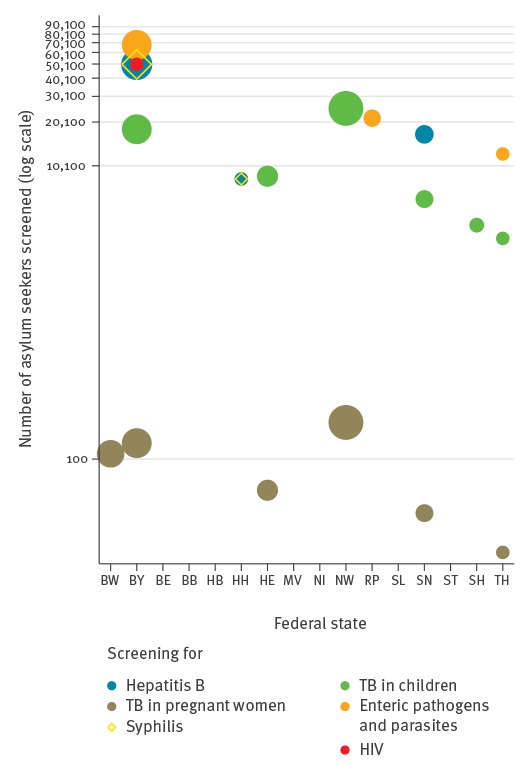
Weighted scatter plot of the estimated number of asylum seekers affected by mandatory diagnostic tests, by federal state, Germany, 2015 (n = 441,899 first-time applicant asylum seekers)

A considerable proportion of individuals were also affected by mandatory screening for syphilis (13.1%) and HIV (11.3%). Because of the weighted allocation of asylum seekers based on administrative quota, the relative and absolute number of asylum seekers affected by mandatory testing varied depending on the number of federal states conducting respective screenings (Table 1), as well as on the relative quota weight of respective federal states (Figure 1).

Plot size: The observations are weighted according to the administrative quota (Königsteiner Schlüssel [11]) reflecting the proportion of asylum seekers received by respective states. The chest X-ray for TB in adults was conducted by all federal states and is not illustrated here.

BB: Brandenburg; BE: Berlin; BW: Baden-Württemberg; BY: Bavaria; HB: Bremen; HE: Hesse; HH: Hamburg; MV: Mecklenburg-Western Pomerania; NI: Lower Saxony; NW: North Rhine-Westphalia; RP: Rhineland-Palatinate; SH: Schleswig-Holstein; SL: Saarland; SN: Saxony; ST: Saxony-Anhalt; TH: Thuringia.

### Economic evaluation of costs relative to screening outcomes

Using statutory insurance fees, the estimated total cost for all compulsory screening measures in 2015 amounted to 10.3 million EUR. Using statutory insurance fees, the highest estimated total cost for a medical screening measure was ca 5.3 million EUR for the initial chest X-ray in asylum seekers ≥16 years of age, followed by the costs for IGRAs to rule out TB in children aged 5–15 years (2.0 million EUR), the costs for hepatitis B screening (1.9 million EUR) and the costs for stool examinations (0.8 million EUR) (Table 2).

**Table 2:** Estimated unit costs, total costs and costs per identified case for the initial screening measures of asylum seekers, Germany, 2015 (n = 441,899 first-time applicant asylum seekers)

Screening measure/ Diagnostic test	Unit costs		Eligible population	Population screened (n)	Total costs		Estimated yield (%)	Estimated cases identified (n)	Costs per identified case	
	Statutory insurance fees^a^ (EUR)	Private fees^b^ (EUR)			Statutory insurance fees^a^ (EUR)	Private fees^b^ (EUR)			Statutory insurance fees^a^ (EUR)	Private fees^b^ (EUR)
**Hepatitis B**										
HBs antigen	5.5	14.6	≥ 16 years of age	74,568	ND	ND	NA	ND	ND	ND
HBc IgM antibody	8.5	17.5			ND	ND	NA	ND	ND	ND
HBe antigen	10.9	14.6			ND	ND	NA	ND	ND	ND
Total	24.9	46.7			1,856,743	3,482,340	Positive seroprevalence: 4.0 [20]	2,982.7	622.5	1,167.5
**Syphilis**										
TPHA/TPPA test, immunoassay	4.6	5.3	≥ 16 years of age	58,002	266,809	307,411	Positive search-test: 0.4 [27]	232.0	1,150.0	1,325.0
**Tuberculosis screening**										
Tuberculosis skin test	0.9	5.3	0–5 years of age	30,993	27,894	164,264	NA	ND	ND	ND
Interferon gamma release assay	58.8	52.5	6–15 years and pregnant women ≥ 16 years of age	34,048	2,002,022	1,787,520	NA	ND	ND	ND
Chest X-ray	15.9	16.3	≥ 16 years of age	323,958	5,150,932	5,280,515	Active TB cases: 0.3 [21]	971.9	5,300.0	5,433.2
**HIV**										
HIV-1 or HIV-1/2 antibody immunoassay (search test)	4.1	17.5	≥ 16 years of age	49,793	204,151	871,372	Positive search-test: 0.79 [20]	398.3	512.6	2,187.7
HIV-1, HIV-2 antibody Western blot (confirmation test)	53.6	46.6	≥ 16 years of age and tested positive in search test	393	21,065	18,332	ND	ND	-	-
Total	–	–	–	49,793	225,216	889,705	Confirmed HIV positive cases: 0.6 [20]	298.8	753.7	2,977.6
**Stool examination**										
Microbiological stool examination (3 plates) on enteropathogens	8.0	14.6	All ages	101,255	810,040	1,478,319	*Shigella* spp.: 0.01 [19,20]	15.9	80,202.0	146,368.2
							*Salmonella* spp.: 0.1 [19,20]	158.6	7,996.4	14,593.5
							Intestinal parasites: 3.5 [20]	5,550.7	228.6	417.1

–: not applicable; EUR: euros; NA: no reliable data available; ND: not determined due to lack of reliable data; TB: tuberculosis; TPHA: Treponema pallidum haemagglutination assay; TPPA: Treponema pallidum particle agglutination.

^a^ Statutory insurance fees refer to fees of the unified reimbursement scheme of the association of statutory sickness fund physicians (Einheitlicher Bewertungsmaßstab, EBM).

^b^ Private fees refer to the reimbursement scheme of private physician services (Gebührenordnung der Ärzte, GOÄ).

Considering the estimated yield of screening for respective diseases, the costs of the initial screening test per identified case were highest for *Shigella* spp. (ca 80,200 EUR), followed by *Salmonella* spp. (ca 8,000 EUR) and TB in adults (5,300 EUR). Due to a lack of yield estimates for TB screening in pregnant women and children seeking asylum, we were not able to estimate the corresponding costs per case for these tests.

The estimated total costs using private fees was 13.4 million EUR and thus ca 1.3 times the estimated total cost based on statutory insurance fees (Table 2). The costs per identified case in the private reimbursement scheme increased by 300% for a case of HIV (3.95-fold), 90% for a case of hepatitis B (1.88-fold), 80% for a case of *Shigella* spp., *Salmonella* spp. or intestinal parasites (1.82-fold), and ca 15% for a case of syphilis (1.15-fold) (Table 2).

### Effect of screening policies on differences in costs between states

The estimated total costs of compulsory screening correspond to average costs of more than 659,000 EUR (95% CI: 192,958–1,126,190) per federal state using statutory insurance fees, but the estimates varied considerably depending on the screening policy in the respective categories (Table 3).

**Table 3:** Mean total cost and mean per capita costs of medical screening of asylum seekers by category of screening policy and reimbursement scheme, Germany, 2015 (n = 441,899 first-time applicant asylum seekers)

	Statutory insurance fees		Private fees		
Screening policy category^a^	Mean total costs (EUR)	95% CI	Mean total costs (EUR)	95% CI	Number of federal states in category
**Germany**	659,574	192,958–1,126,190	1,118,890	74,459–2,163,322	16
**Category A**	180,118	73,602–286,635	185,343	75,737–294,948	7
**Category B**	871,739	314,527–1,428,952	876,500	320,388–1,432,612	4
**Category C**	1,161,080	112,042–2,210,118	2,619,770	138,684–5,100,855	5
Screening policy category^a^	Mean per capita costs (EUR)	95% CI	Mean per capita costs (EUR)	95% CI	Number of federal states in category
**Germany**	20.5	14.3–26.7	33.8	15.8–51.7	16
**Category A**	11.6	11.4–11.8	12.0	11.8–12.1	7
**Category B**	17.9	14.2–21.5	18.0	14.4–21.5	4
**Category C**	35.1	24.0–46.1	77.0	41.7–112.3	5

CI: confidence interval.

^a^ Federal states were categorised into three groups according to their screening policies. Category A: Federal states exclusively screening for tuberculosis (TB) among asylum seekers ≥ 16 years of age (Brandenburg, Berlin, Bremen, Mecklenburg-Western Pomerania, Lower Saxony, Saarland and Saxony-Anhalt); Category B: Federal states performing extended TB screening in children < 16 years of age and/or pregnant women in addition to Category A (Baden-Württemberg, Hesse, North Rhine-Westphalia and Schleswig-Holstein); Category C: Federal states performing any other extended screening measures in addition to measures in Category A or B (Bavaria, Hamburg, Rhineland-Palatinate, Saxony and Thuringia).

**Figure 2:**
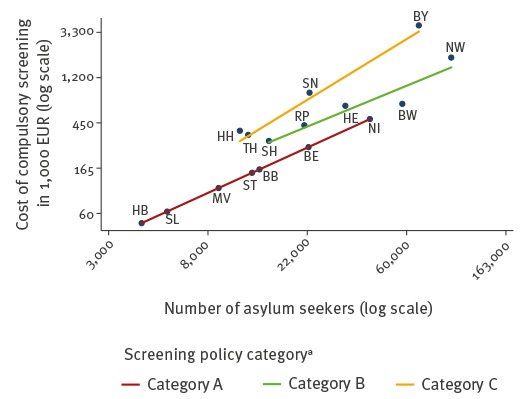
Scatter plot of estimated total costs of medical screening measures by number of asylum seekers and category of screening policy, Germany, 2015 (n = 441,899 first-time applicant asylum seekers)

BB: Brandenburg; BE: Berlin; BW: Baden-Württemberg; BY: Bavaria; HB: Bremen; HE: Hesse; HH: Hamburg; MV: Mecklenburg-Western Pomerania; NI: Lower Saxony; NW: North Rhine-Westphalia; RP: Rhineland-Palatinate; SH: Schleswig-Holstein; SL: Saarland; SN: Saxony; ST: Saxony-Anhalt; TH: Thuringia.

^a^ Federal states were categorised into three groups according to their screening policies. Category A: Federal states exclusively screening for tuberculosis (TB) among adult asylum seekers ≥ 16 years of age; Category B: Federal states performing extended TB screening in children < 16 years of age and/or pregnant women in addition to Category A; Category C: Federal states performing any other extended screening measures in addition to measures in Category A or B.

Estimated total costs for medical screening measures showed a positive correlation with the total number of asylum seekers, but also a clear relationship with the type of screening policy (Figure 2).

Mean per capita costs of medical screening of asylum seekers were higher in federal states with extended TB screening policies and in federal states with compulsory screening for sexually transmitted infections (STIs) and/or intestinal infections (Table 3).

**Figure 3:**
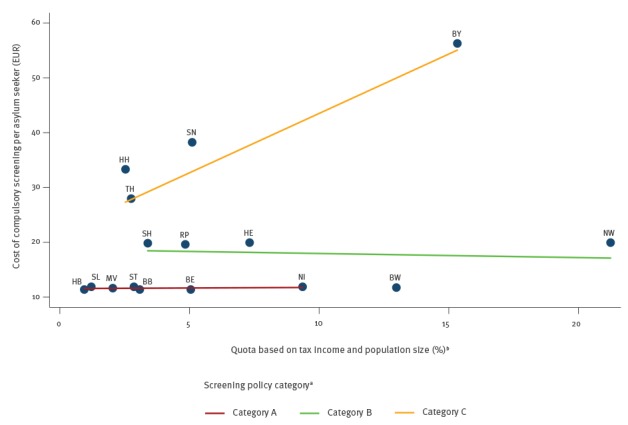
Scatter plot of estimated per capita costs of medical screening of asylum seekers by the socioeconomic strength of federal states and category of screening policy, Germany, 2015 (n = 441,899 first-time applicant asylum seekers)

BB: Brandenburg; BE: Berlin; BW: Baden-Württemberg; BY: Bavaria; HB: Bremen; HE: Hesse; HH: Hamburg; MV: Mecklenburg-Western Pomerania; NI: Lower Saxony; NW: North Rhine-Westphalia; RP: Rhineland-Palatinate; SH: Schleswig-Holstein; SL: Saarland; SN: Saxony; ST: Saxony-Anhalt; TH: Thuringia.

^a^ Federal states were categorised into three groups according to their screening policies. Category: A: Federal *s*tates exclusively screening for TB among asylum seekers ≥ 16 years of age; Category B: Federal states performing extended TB screening in children < 16 years of age and/or pregnant women in addition to Category A; Category C: Federal states performing any other extended screening measures in addition to measures in Category A or B.

^b^ Administrative quota for the year 2015 taken from the Joint Science Conference [11].

As a consequence, federal states with the same level of socioeconomic strength (measured by tax income and population size and implemented with the administrative allocation quota [12] had different per capita costs for medical screening of asylum seekers depending on their screening policies (Figure 3).

The per capita screening costs in federal states with TB screening of children and/or pregnant women (i.e. Category B) were significantly higher (p = 0.006), on average 6.2 EUR (i.e. 50%) higher, compared with the per capita screening costs in federal states limiting screening to asylum seekers ≥ 16 years of age (i.e. Category A) (Table 4).

**Table 4:** Effect of screening policies on estimated per capita cost of screening of asylum seekers obtained by linear regression analysis using statutory insurance fees, Germany, 2015 (n = 16 federal states)

Per capita screening cost differences vs reference group (Category A)^a^	Per capita screening cost (EUR)	SE^b^	p value^c^	95% CI	R-squared	F-statistic (Model df)
**Absolute between-group difference**						
States performing extended TB screening (Category B)	6.23	1.97	0.006	2.05–10.43	0.67	12.46 (2, 15)
States performing any other extended screening measures (Category C)	23.45	6.09	0.002	10.48–36.43		
**Relative between-group difference (rate ratios)^d^**						
States performing extended TB screening (Category B)	1.50	0.13	0.006	1.14–1.97	0.80	23.43 (2, 15)
States performing any other extended screening measures (Category C)	2.84	0.17	< 0.0001	1.96–4.10		

CI: confidence interval; df: degrees of freedom; SE: standard error.

^a^ Category A (reference group): Federal states exclusively performing screening for TB among asylum seekers ≥ 16 years of age; Category B: Federal states performing extended TB screening in children < 16 years of age and/or pregnant women in addition to Category A; Category C: Federal states performing any other extended screening measures in addition to measures in Category A or B.

^b^ Standard errors: adjusted for n = 16 clusters at federal state level.

^c^ p value of a t-test, bold figures indicate statistical significance below the 0.05 level.

^d^ Rate ratios were calculated by the exponential of the regression estimates (exp(*β*_1_)) obtained from linear regression analysis using the natural logarithm of per capita screening cost (EUR) as outcome.

The absolute and relative differences in per capita screening costs increased to 23.5 EUR, i.e. to 184%, when comparing states with extended screening for diseases besides TB (Category C) with the reference federal states (Category A) (Table 4).

Using private fees, we found a significant difference (p = 0.005) in per capita costs of screening between states with extended screening (Category C) and those with basic TB screening (Category A) of 65.0 EUR (95% CI: 22.7–107.2) in absolute terms and more than 400% (RR: 5.4; 95% CI: 2.8–10.5) in relative terms.

## Discussion

This study analysed the compulsory medical screening of asylum seekers in Germany and provided estimates of the coverage and costs relative to expected yields. By listing and comparing state-level policies, we generated a comprehensive nation-wide overview of the content of screening programmes. Our study reveals substantial heterogeneity with respect to the range of compulsory screening tests stipulated by state policies and illustrates how this affects the proportion of asylum seekers screened as a consequence of the quota-based allocation system. The heterogeneity in screening policies leads to different economic impacts with respect to the distribution of the costs of receiving asylum seekers; federal states with the same level of economic strength mobilise different amounts of resources per asylum seeker for medical screening implementation. A high number and proportion of newly arrived asylum seekers were affected by compulsory screening for STIs (hepatitis B, syphilis or HIV) and stool examinations, in addition to the mandatory chest X-ray performed in asylum seekers ≥ 16 years of age. This resulted in total costs for Germany of more than 10 million EUR in 2015, a conservative, lower-bound estimate assuming full reimbursement according to statutory insurance fees. The sensitivity analysis showed that the total costs at the upper-bound using private fees could be up to 30% higher, which further argues in favour of the introduction of electronic health cards for asylum seekers and their integration into the regular healthcare system in Germany [23,24]. We found high costs of medical screening relative to expected yields, raising questions on the cost-effectiveness of screening for some of the pathogens or diseases. By far the highest cost per identified case with respect to the initial screening test was found for *Shigella* spp. (> 80,000 EUR), followed by *Salmonella* spp. and TB (each > 5,000 EUR), syphilis (> 1,000 EUR), as well as HIV (> 700 EUR) and hepatitis B infections (> 600 EUR). Considering the co-existing private fee arrangements for conducting the medical screenings, the benefits of screening relative to programme costs are likely to be even lower, especially for infections with HIV, hepatitis B and enteropathogens.

In Germany, a large population of asymptomatic asylum seekers undergoes compulsory screening for a wide range of diseases either performed by public health agencies at federal state level or other healthcare providers. The underlying rationale is not only the protection of the asylum seekers, but also that of the host population. To assess the potential benefits and problems of screening programmes, well-established criteria that span public health, medical and normative/ethical perspectives must be applied [7].

Active TB can be considered an important condition to screen for from a public health perspective [25,26], but compulsory screening for sexually transmitted infections (STIs) and intestinal parasites among asymptomatic populations may not be beneficial and should be well reasoned considering the entailed cost per identified case. From the perspective of individual medical care, early diagnosis of these conditions in reception centres, including referral to specialists and initiation of high-quality care, is very desirable [6]. However, this is not always possible in practice. One of the few evaluations on follow-up of screening programmes provides a pertinent example: among 31,660 asylum seekers screened for syphilis in Hamburg, 236 (0.7%) had positive results in the first test, but 94% of these were not followed up for the second (confirmatory) test. As a consequence, the public health services ceased screening for syphilis in April 2016 [27]. Likewise, Bavaria ceased compulsory medical screening for syphilis in September 2015 and for *Shigella spp.* and *Salmonella spp.* in October 2015. Some federal states have ceased hepatitis B screening several years ago due to limited capacities to give appropriate follow-up to both negative and positive diagnostic results [13].

Alternatives to compulsory extensive screening in the heterogeneous group of asylum seekers may include good and low threshold access to primary medical care [28] as well as targeted screening for at risk groups, e.g. based on country of origin, individual risk factors and clinical parameters [29]. Entitlements and access to healthcare for asylum seekers are restricted in Germany based on the argument of resource constraints [30,31]. Some studies demonstrated that highly prevalent mental health conditions like depression, anxiety and post-traumatic stress disorder in asylum seekers [32-34] remain poorly addressed [35]. Ceasing extensive screening for infectious diseases, except for TB, in the more than 120,000 asylum seekers in all five states in Category C would have released an estimated 3.1 million EUR. This would have provided the opportunity of investing them in the provision of mental health services in reception centres at a ratio of one psychologist per 2,000 asylum seekers per year (at a monthly salary of ca 4,000 EUR [36]).

The main strengths of this study are (i) that it is a comprehensive overview of the wide range of compulsory screening for infectious diseases in one of the largest recipient countries of asylum seekers and (ii) that it uses nationally reported yields of screening in asylum seekers and routine health system market values to generate a first-time estimate of the costs of screening measures in relation to their expected yields. We illustrated the high between-state variance in screening policies and quantified the differences in per capita costs between states.

The main limitation of our study is that the estimates of total costs and costs per identified case (i) are exclusively based on direct costs for the initial diagnostic tests and (ii) rely on the assumption of nation-wide use of statutory insurance fees. However, only North Rhine-Westphalia, the largest recipient state, has implemented a state-wide reimbursement scheme for service providers according to statutory insurance fees. Thus, the true total cost of compulsory screening programmes is likely higher due to direct and indirect healthcare costs beyond laboratory measures, the possibility of repeat testing the same individual and the heterogeneity of service provider re-imbursement between and within a federal state according to the type of fees. Another possible source of underestimation is the use of the number of first-time asylum applicants in 2015 to determine the total population of individuals screened. Although 441,899 first-time asylum applications were received [16], the number of individuals entering the country registered by BAMF was more than 800,000 [37], though this number includes the possibility of double-registration by immigration authorities. Our estimates are thus very conservative.

Furthermore, the economic evaluation was not designed to decide whether, for example, screening for hepatitis B should be preferred over screening for TB. Such an analysis would need comparable measures of screening outcome, such as quality-adjusted life years gained or mortality avoided. It was also not designed to compare the cost-effectiveness of screening for certain diseases vs no screening since this would require (i) nation-wide information, disaggregated by residence status, on the number of cases of a certain infectious disease identified in routine care and (ii) information on whether the screening led to follow-up care. Such data are not available due to limitations in the health information system and the lack of integrated quality assurance in screening policies. The available data did not allow for dynamic decision modelling, as this would require more data on the diagnostic precision of tests and test alternatives in the population under study, disease prevalence in asylum seekers of different age groups and countries of origin, the number of cases that would have been identified without screening, and information on the course of the disease with and without screening. Such data are not available for asylum seekers and are urgently needed [14].

Further research is needed to assess the benefits and weaknesses of compulsory screening for infectious diseases in asylum seekers. The high estimated costs of medical screening in this study, especially for diseases with a low yield, call for the development of evidence-based and more targeted approaches to infectious disease screening in this vulnerable population group.
